# Proangiogenic role of circRNA‐007371 in liver fibrosis

**DOI:** 10.1111/cpr.13432

**Published:** 2023-02-28

**Authors:** Chong Zhao, Shuaijie Qian, Yang Tai, Yangkun Guo, Chengwei Tang, Zhiyin Huang, Jinhang Gao

**Affiliations:** ^1^ Lab of Gastroenterology and Hepatology West China Hospital, Sichuan University Chengdu China; ^2^ Department of Gastroenterology West China Hospital, Sichuan University Chengdu China

## Abstract

Circular RNAs (circRNAs) are crucially involved in cancers as competing endogenous RNA (ceRNA) or microRNA (miRNA) sponges. However, the function and mechanism of circRNAs in liver fibrosis remain unknown and are the focus of this study. Murine fibrotic models were induced by thioacetamide (TAA) or carbon tetrachloride (CCl_4_). Increased angiogenesis is accompanied by liver fibrosis in TAA‐ and CCl_4_‐induced murine fibrotic livers. circRNA microarray and argonaute 2 (AGO2)‐RNA immunoprecipitation (RIP) sequencing (AGO2‐RIP sequencing) were performed in murine livers to screen for functional circRNAs. Compared to control livers, 86 differentially expressed circRNAs were obtained in TAA‐induced murine fibrotic livers using circRNA microarray. In addition, 551 circRNAs were explored by AGO2‐RIP sequencing of murine fibrotic livers. The circRNA‐007371 was then selected and verified for back‐spliced junction, resistance to RNase R, and loop formation. In vitro, murine hemangioendothelioma endothelial (EOMA) cells were transfected with circRNA‐007371 overexpressing plasmid or empty plasmid. circRNA‐007371 overexpression promoted tube formation, migration, and cell proliferation of EOMA cells. RNA sequencing and miRNA sequencing were then performed to explore the mechanism of the proangiogenic effects of circRNA‐007371. circRNA‐007371 promotes liver fibrosis via miRNA sponges or ceRNA mechanisms. *Stag1*, the parent gene of circRNA‐007371, may play a significant role in proangiogenic progression. In conclusion, circRNA‐007371 enhances angiogenesis via a miRNA sponge mechanism in liver fibrosis. The antiangiogenic effect of circRNA‐007371 inhibition may provide a new strategy for treating patients with liver cirrhosis.

## Graphical abstract

The expression profiling of circRNA with ceRNA function in the murine fibrotic liver was displayed. A new circRNA, circRNA‐007371, which contributed to the pathogenesis of liver fibrosis, was verified. A new mechanism by which circRNA‐007371 promoted angiogenesis as a miRNA sponge was discovered.

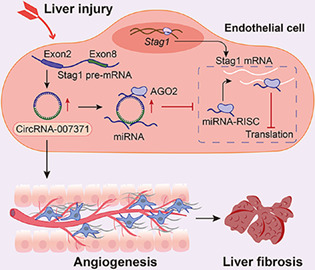

## 1 INTRODUCTION

Liver cirrhosis is a result of the progression of chronic liver disease (CLD), which causes serious complications, including ascites, variceal haemorrhage, hepatic encephalopathy, and an increased risk for hepatocellular carcinoma (HCC).^1^, ^2^, ^3^ CLD and liver cirrhosis lead to a high disease burden, with approximately 2 million deaths worldwide each year.^4^, ^5^ Multiple pathophysiological mechanisms participate in fibrogenesis during chronic liver injury, including angiocrine signalling,^6^, ^7^, ^8^, ^9^ hepatic stellate cell (HSC) activation,^10^, ^11^ and the inflammatory response.^6^, ^12^ Although widely studied, no effective medical management is available to reverse fibrosis in patients with liver cirrhosis. Therefore, more investigations are urgently needed to clarify the mechanism of liver cirrhosis to explore novel therapeutic targets.

Angiogenesis is defined as new blood vessel formation from the preexisting vasculature.^13^ Pathological angiogenesis leads to dysfunctional liver sinusoidal endothelial cells (LSECs) in the transport and exchange of oxygen and nutrients, enhances inflammation aggravation, and raises vascular resistance, eventually leading to the progression of liver cirrhosis.^13^, ^14^ Traditionally, the vascular endothelial growth factor (VEGF) and hypoxia‐inducible factor (HIF) pathways are master regulators in pathological angiogenesis,^13^, ^15^, ^16^ and angiocrine factors in LSECs contribute to pathological angiogenesis.^17^, ^18^ However, it remains unclear whether other regulators also contribute to pathological angiogenesis.

As endogenous noncoding and covalently closed RNAs, circular RNAs (circRNAs) are produced by pre‐messenger RNA (mRNA) through back‐splicing.^19^ circRNAs play crucial roles in liver diseases as microRNA (miRNA) sponges or competing endogenous RNAs (ceRNAs).^19^, ^20^ By binding to the parent mRNA, miRNAs combine with the argonaute (AGO) protein to form an RNA‐induced silencing complex (RISC), which leads to mRNA degradation, translation blockade, and parent gene expression inhibition.^21^, ^22^ However, as miRNA sponges or ceRNAs, circRNAs compete with the target sites of miRNA–RISC, which further relieves its inhibitory effects on parent genes.^21^ circRNAs can regulate HSC activation^23^, ^24^ and quiescence^25^, ^26^ through miRNA sponges in liver fibrosis. Previous studies have emphasized the crucial effects of circRNAs in angiogenesis in HCC.^27^, ^28^ Nevertheless, it remains unclear that the function and potential mechanism of circRNAs in the angiogenesis of liver fibrosis. circRNAs can compete with miRNAs to abrogate the inhibitory effects of miRNA on parent genes. Moreover, miRNAs participate in angiogenesis in the context of HCC^29^, ^30^ and liver fibrosis.^31^ Whether circRNAs can regulate angiogenesis in liver fibrosis as miRNA sponges or ceRNAs remains unexplored.

This study aimed to detect the proangiogenic function and potential mechanism of circRNAs in liver fibrosis. Increased angiogenesis is accomplished with liver fibrosis in murine fibrotic models. As screened by circRNA microarray and AGO2 RNA immunoprecipitation (RIP) sequencing (AGO2‐RIP sequencing) in thioacetamide (TAA)‐induced murine fibrotic livers, circRNA‐007371 was chosen as the target circRNA. circRNA‐007371 is characterized by back‐splicing of exon 2 and exon 8 of stromal antigen 1 (*Stag1*). In vitro, circRNA‐007371 overexpression promoted cell migration, vessel formation, and cell proliferation in murine hemangioendothelioma endothelial (EOMA) cells. Then, the genes, miRNAs, and signalling pathways affected by circRNA‐007371 were explored in circRNA‐007371 overexpressing EOMA cells using mRNA sequencing and miRNA sequencing. Together, the angiogenic role of circRNA‐007371 might promote liver fibrosis via miRNA sponges or ceRNA mechanisms.

## 2 MATERIALS AND METHODS

### 2.1 Murine models for liver fibrosis

The animal procedures were approved and conducted by the Animal Use and Care Committee of West China Hospital, Sichuan University. All animals were freely fed food and water under 12‐h light/dark cycles.

Wild‐type male C57BL/6J mice 8–12 weeks old were obtained from GemPharmate Co. Ltd. The mice were randomly assigned to four groups with eight animals in each group, including the saline, TAA, olive oil (OO), and carbon tetrachloride (CCl_4_) groups. For the murine fibrotic models, mice received an intraperitoneal (ip) injection of TAA (200 mg/kg, Sigma #163678) twice a week for 8 weeks or CCl_4_ (4 μL/g with 1:3 diluted in OO, RHAWN #R033168) twice a week for 6 weeks. For the control groups, mice were ip injected with normal saline or OO. The mice were sacrificed with an overdose of sodium pentobarbital 48 h after the last injection.

### 2.2 circRNA microarray and data analysis

Five liver tissues from the TAA group and five liver tissues from the saline group were sent to the circRNA microarray. The circRNA microarray procedure and data analysis were performed by KangCheng Biotech. The raw data of the circRNA microarray were deposited in the NCBI Gene Expression Omnibus (GEO; GSE218574).

### 2.3 Anti‐AGO2‐RIP sequencing and data analysis

Anti‐AGO2‐RIP followed by RNA sequencing (AGO2‐RIP sequencing) was used to explore the expression of RNAs that bind to AGO2. Liver tissues from the TAA group were utilized for AGO2‐RIP sequencing. AGO2‐RIP sequencing was performed by Shanghai Cloud‐Seq Biotech. The raw data of AGO2‐RIP sequencing were deposited in the NCBI GEO (GSE218577).

### 2.4 mRNA sequencing and miRNA sequencing

The control and circRNA‐007371 overexpressing EOMA cells were subjected to mRNA and miRNA sequencing. The RNA sequencing process and data analysis were performed by Novogene. The raw mRNA sequencing (GSE218578) and miRNA sequencing (GSE218579) data have been deposited in the NCBI GEO.

### 2.5 Statistical analysis

All data are shown as the mean ± standard deviation and were analysed using GraphPad software (version 9; GraphPad Software Inc.). Then, *t*‐tests and one‐way ANOVA followed by the Bonferroni post hoc test were utilized for statistical analysis. A significant difference was defined as *p* < 0.05.

The detailed methods are listed in the Supporting Information, including the histological study, immunohistochemical staining, immunofluorescence (IF), Western blot (WB), circRNA microarray, AGO2‐RIP sequencing, mRNA sequencing, miRNA sequencing, quantitative RT‐PCR (qPCR), agarose gel electrophoresis, RNase R treatment assay, cell culture, circRNA‐007371 overexpression, wound healing assay, and tube formation assay.

## 3 RESULTS

### 3.1 Accomplishment of angiogenesis with liver fibrosis in mice

**FIGURE 1:**
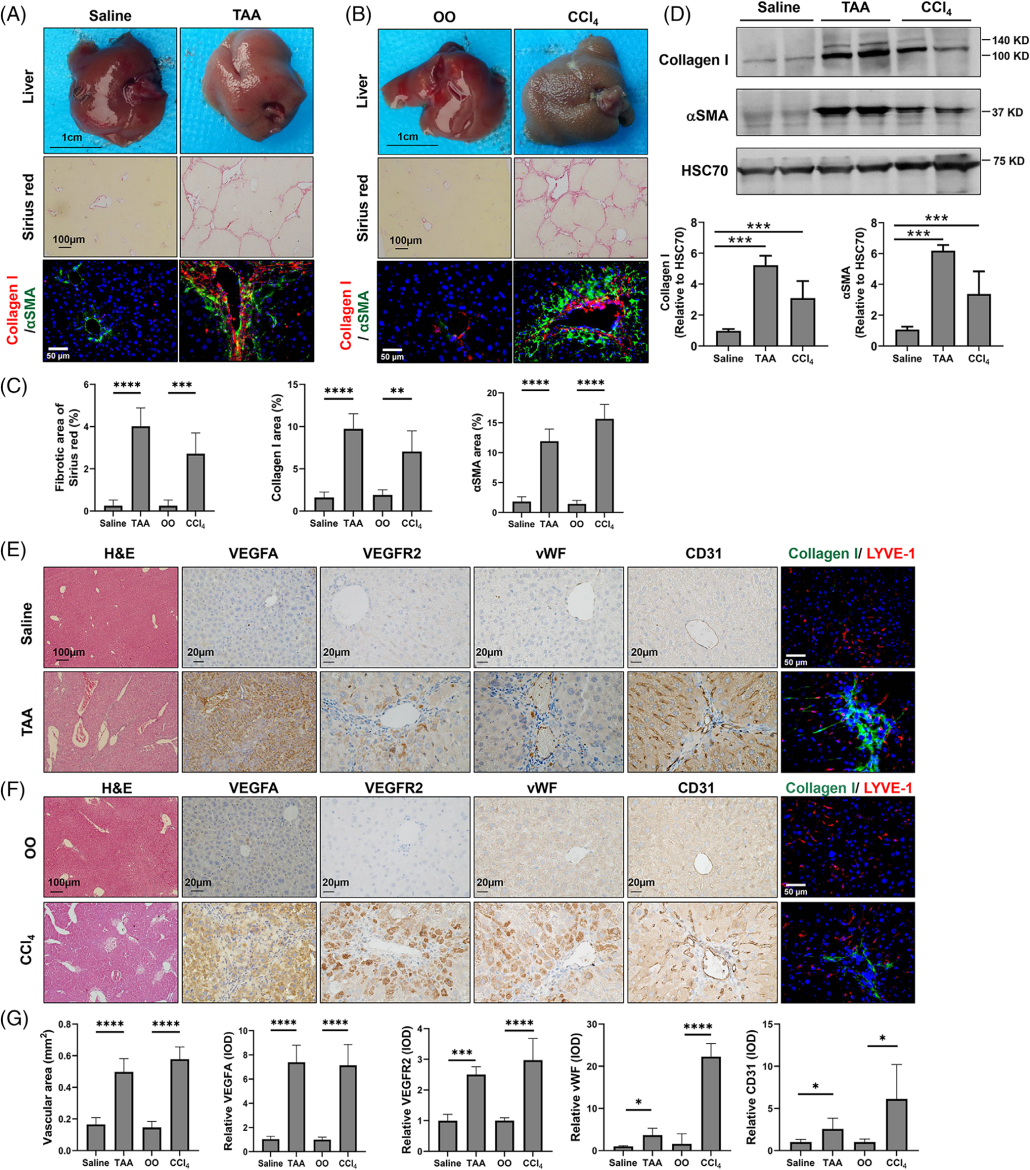
Accomplishment of angiogenesis with liver fibrosis in mice. Wild‐type C57BL/6J mice were intraperitoneal injections with olive oil (OO) or carbon tetrachloride (CCl_4_) for 6 weeks (*n* = 8/group) or saline or thioacetamide (TAA) for 8 weeks (*n* = 8/group). (A–D) Liver fibrosis was grossly observed and analysed by Sirius red staining and colocalization of collagen I and αSMA by immunofluorescence (IF) in the TAA‐ (A, C) or CCl_4_‐induced murine models (B, C). The protein levels of collagen I and αSMA were quantified by Western blot (D). (E–G) Hepatic angiogenesis was analysed by haemotoxylin and eosin (H&E) staining, vascular area of H&E staining, immunohistochemical staining for VEGFA, VEGFR2, von Willebrand factor (vWF), and CD31, and immunofluorescence for costaining of LYVE‐1 and collagen I in the TAA‐ (E, G) or CCl_4_‐induced murine models (F, G). *p < 0.05, **p < 0.01, ***p < 0.001, ****p < 0.0001.

Angiogenesis is defined as new blood vessel formation from the preexisting vasculature.^32^ As the VEGF‐mediated angiogenesis signalling pathway is predicted to be involved in nonalcoholic steatohepatitis‐ and hepatitis B virus‐related liver cirrhosis,^33^, ^34^ we first determined angiogenesis in murine fibrotic models induced by TAA and CCl_4_. As expected, compared to corresponding controls, murine livers with extensive fibrosis were observed in gross and Sirius red staining of the TAA and CCl_4_ groups (Figure 1A–C). Consistently, significantly increased collagen deposition and HSC activation were confirmed as quantified by IF and WB of collagen I and αSMA in the TAA and CCl_4_ groups compared to the control groups (Figure 1A–D). We next assessed angiogenesis in the murine fibrotic livers. Compared to controls, angiogenesis determined by the vascular area of haemotoxylin and eosin staining was significantly enhanced in the TAA and CCl_4_ groups (Figure 1E–G). The proangiogenic protein levels of VEGFA and VEGF receptor 2 (VEGFR2) were also significantly increased in the TAA and CCl_4_ groups compared to the control groups (Figure 1E–G). Furthermore, the angiogenesis marker proteins von Willebrand factor and CD31 were significantly raised in the TAA and CCl_4_ groups compared to the control groups (Figure 1E–G). Neovascularization was mainly distributed in the fibrotic septa of the murine livers in both the TAA and CCl_4_ groups as determined by colocalization of LYVE‐1 and collagen I (Figure 1E,F). In summary, increased angiogenesis is accomplished with liver fibrosis in TAA‐ and CCl_4_‐induced murine fibrotic livers.

### 3.2 Expression profiling and potential functions of circRNAs in murine fibrotic livers

**FIGURE 2:**
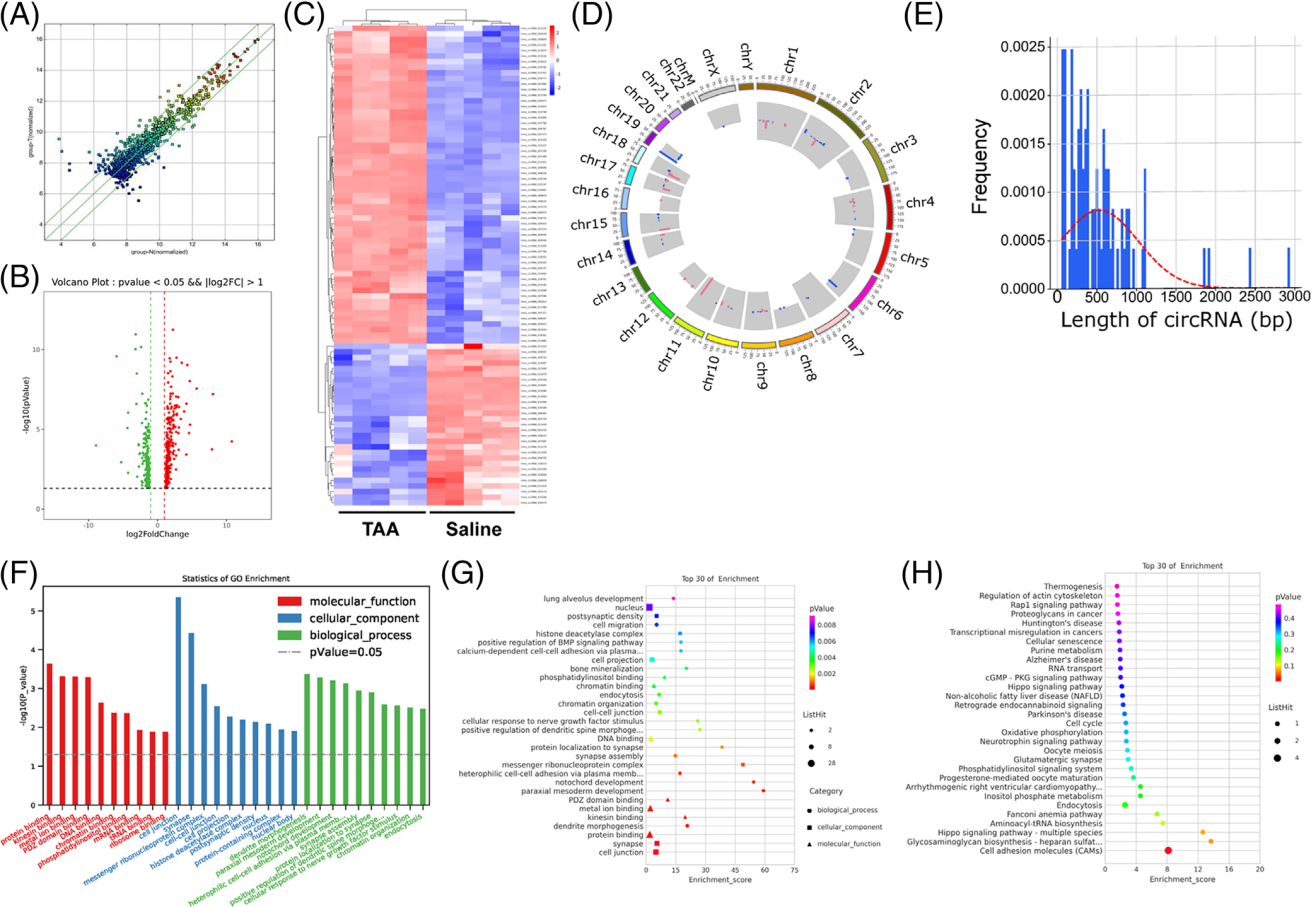
Expression profiling and potential functions of circular RNAs (circRNAs) in murine fibrotic livers. circRNA microarray was performed in livers from the saline or thioacetamide (TAA) groups (*n* = 5/group). (A–E) Scatter plots (A), volcano plots (B), heatmap (C), distribution on mouse chromosomes (D), and frequency length (E) of differentially expressed circRNAs (DEcircRNAs) were shown. (F–H) The Gene Ontology analysis displayed with bar (F) or bubble plot (G) and Kyoto Encyclopedia of Genes and Genomes pathway analysis (H) of the parent genes of DEcircRNAs were revealed.

circRNAs play crucial roles in the pathogenesis of angiogenesis and liver diseases.^19^ To explore hepatic circRNA expression profiling in liver fibrosis, livers from control mice and TAA‐induced liver fibrosis mice were subjected to circRNA microarray analysis. As shown by the scatter plots, volcano plots, and heatmap of differentially expressed circRNAs (DEcircRNAs; Figure 2A–C), 57 upregulated circRNAs and 29 downregulated circRNAs were detected in TAA‐induced murine fibrotic livers compared to control livers (*p* < 0.05 and fold change > 2.0). These DEcircRNAs were distributed on 19 chromosomes (Figure 2D). The frequency length of DEcircRNAs mainly ranged from 50 to 1300 base pairs (bp) (Figure 2E). Gene Ontology (GO) analysis and Kyoto Encyclopedia of Genes and Genomes (KEGG) pathway analysis were utilized to further understand the function of the parent RNA of these DEcircRNAs. The GO analysis indicated that angiogenesis‐related processes, including endocytosis, cell migration, cell–cell adhesion, and cell junctions, were enriched in fibrotic livers (Figure 2F,G). The top 30 most enriched KEGG pathways of these DEcircRNAs also revealed that angiogenesis‐related processes, for example, thermogenesis, cell adhesion, and endocytosis, were enriched in fibrotic livers (Figure 2H). In summary, we uncovered the expression profiles and potential functions of circRNAs in murine fibrotic livers.

### 3.3 Screening of functional circRNAs in fibrotic murine livers

**FIGURE 3:**
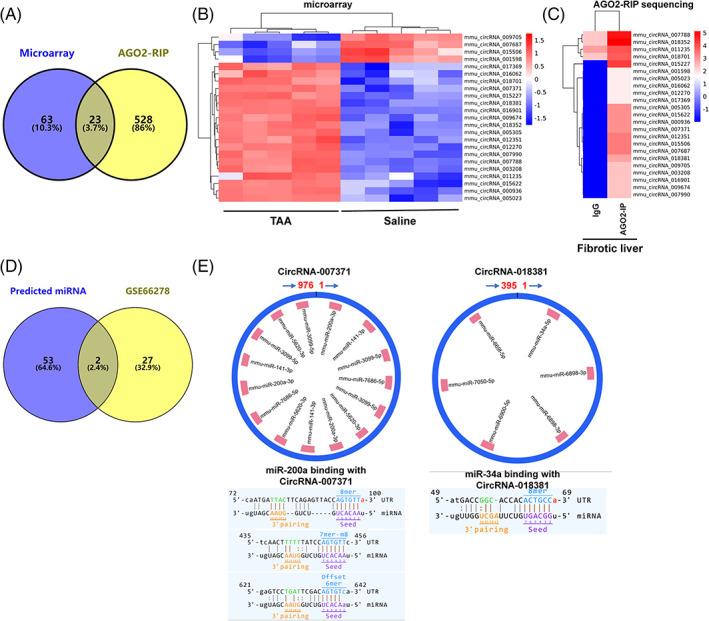
Screening of functional circular RNAs (circRNAs) in fibrotic murine livers. The differentially expressed circRNAs (DEcircRNAs) were retrieved from the circRNA microarray. Argonaute 2‐RNA immunoprecipitation sequencing (AGO2‐RIP) sequencing was utilized in thioacetamide (TAA)‐induced murine fibrotic livers with IgG as a control (*n* = 1/group). (A) Venn diagram displaying the shared circRNAs between DEcircRNAs in the circRNA microarray and AGO2‐RIP sequencing. (B, C) Heatmap showing the expression of 23 shared circRNAs in the circRNA microarray (B) and AGO2‐RIP sequencing (C). (D) The predicted binding microRNAs (miRNAs) of the 23 shared circRNAs were achieved using miRNA target prediction software. The differentially expressed miRNAs (DEmiRNAs) in the murine fibrotic livers induced by (CCl_4_) for 8 weeks were obtained (GSE66278). The Venn diagram displayed the shared miRNAs between predicted binding miRNAs and DEmiRNAs in GSE66278. (E) The structure, miRNA binding sites, and binding sequence of two circRNAs were displayed.

By binding with AGO2, circRNAs can enhance the expression of target genes as ceRNAs or miRNA sponges.^21^ To determine which circRNAs can regulate gene expression as ceRNAs, AGO2‐RIP sequencing was utilized in murine fibrotic livers induced by TAA. In total, 551 circRNAs were explored by AGO2‐RIP sequencing of murine fibrotic livers. The 551 circRNAs obtained from AGO2‐RIP sequencing were merged with 86 DEcircRNAs, and 23 shared circRNAs were displayed (Figure 3A). As shown by the heatmap, these 23 circRNAs were DEcircRNAs in the murine fibrotic liver compared to the control liver with ceRNA potential (Figure 3B,C).

As ceRNAs can act as miRNA sponges, TargetScan and miRanda‐based miRNA target prediction software was applied to predict the miRNAs bound to these 23 circRNAs. In total, 55 miRNAs were found. Furthermore, differentially expressed miRNAs (DEmiRNAs) in the fibrotic murine liver (GSE66278) were retrieved^35^ and merged with the 55 predicted miRNAs (Figure 3D). Two miRNAs, miR‐200a‐5p and miR‐34a‐5p, were eventually identified (Figure 3D). By sequence analysis, miR‐200a‐5p can bind with circRNA‐007371, and miR‐34a‐5p can bind with circRNA‐018381 (Figure 3E). As circRNA‐007371 has more miRNA binding sites (Figure 3E), circRNA‐007371 was selected for further investigation. In summary, circRNA‐007371 may participate in the development of liver fibrosis as a miRNA sponge.

### 3.4 Characterization of circRNA‐007371 as circRNA in murine livers

**FIGURE 4:**
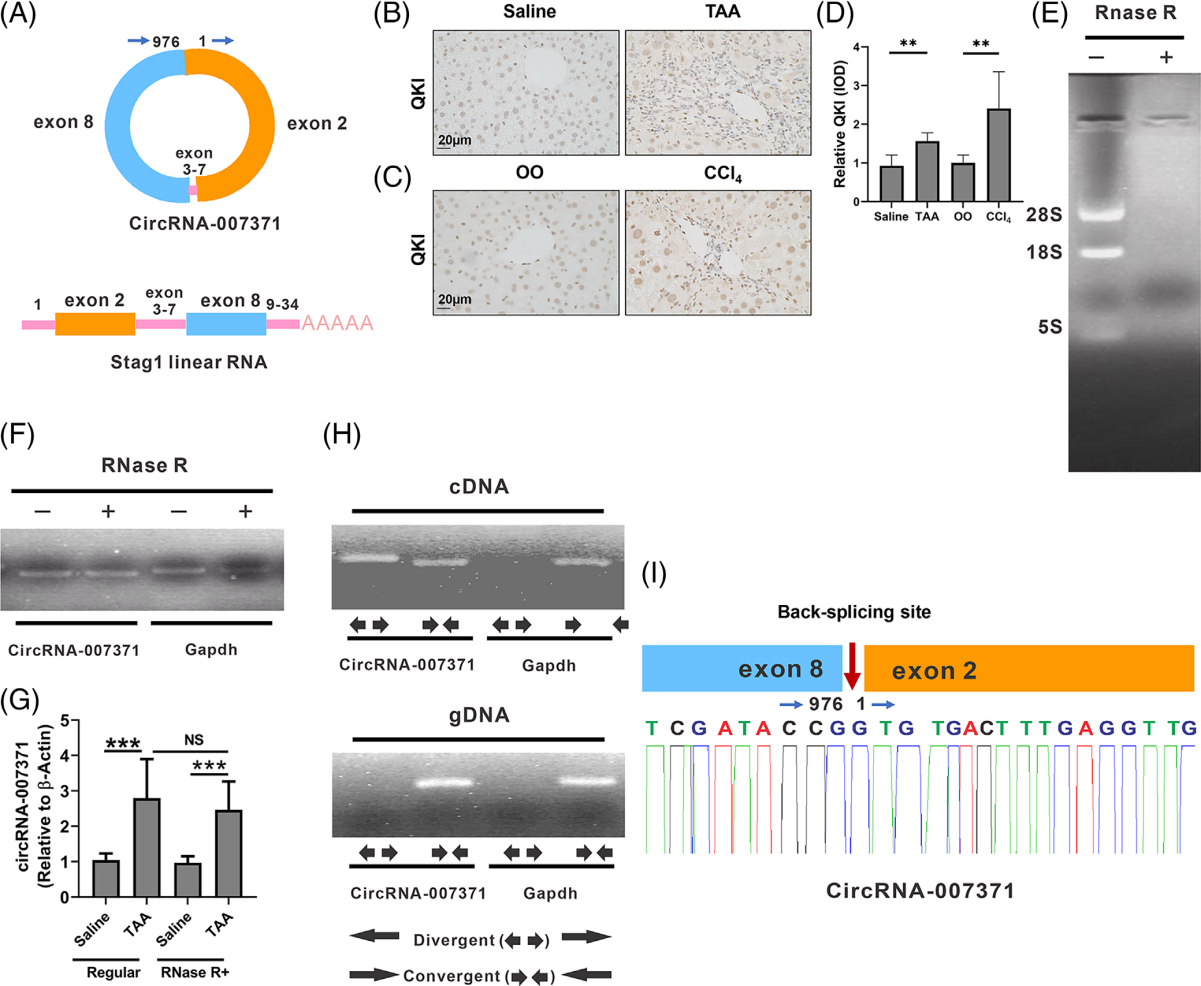
Characterization of circRNA‐007371 as circular RNA (circRNA) in murine livers. (A) Schematic diagram showing the back‐splicing pattern of circRNA‐007371. (B–D) The level of Quaking RNA binding protein (QKI) in fibrotic murine livers was analysed by immunohistochemical staining in the thioacetamide (TAA)‐ (B, D) or carbon tetrachloride (CCl_4_)‐induced murine model (C, D). (E–G) Total RNA was extracted from fibrotic murine livers and treated with or without RNase R. Agarose gel electrophoresis was utilized to detect the RNA (E) and polymerase chain reaction (PCR) products (F). *Gapdh* was used as a control. qPCR was utilized to quantify the level of circRNA‐007371 in the fibrotic and control livers (G), and *β‐actin* was used as a control. *n* = 8/group. (H) PCR‐base agarose gel electrophoresis was applied to evaluate the level of circRNA‐007371 amplified by cDNA and gDNA with divergent and convergent primers. *Gapdh* was used as a control. (I) The back‐spliced junction of circRNA‐007371 was analysed by Sanger sequencing for PCR products obtained with divergent primers. NS, not significant; **p < 0.01; ***p < 0.001.

circRNAs are endogenous noncoding and covalently closed RNAs produced by pre‐mRNA through back‐splicing.^19^ By sequence comparison, we found that circRNA‐007371 is a 976 bp circRNA derived from the parent gene *Stag1* and located at chromosome 9 (NC_000075.7; Figure S1). Presumably, circRNA‐007371 may be back‐spliced from exons 2 and 8 of *Stag1* (Figure 4A). Quaking RNA binding protein (QKI) is an RNA binding protein that can promote the formation of circRNAs.^36^ Compared to controls, the protein level of QKI was increased in TAA‐ and CCl_4_‐induced murine fibrotic livers (Figure 4B–D). circRNA is characterized by high stability to RNase R digestion. RNase R can degrade linear RNA, but circRNAs resist RNase R digestion (Figure 4E). To this end, murine liver total RNA was first treated with RNase R and then reverse transcribed into cloned, reverse‐transcribed mRNA (cDNA), and PCR followed by agarose gel electrophoresis was performed. The linear RNA *Gapdh* was degraded by RNase R, whereas circRNA‐007371 was resistant to RNase R treatment (Figure 4F). Under normal conditions, compared to that in the control livers, the level of circRNA‐007371 in the murine fibrotic livers was significantly increased (Figure 4G). Similar results were obtained when the RNAs were pretreated with RNase R (Figure 4G). These results indicate that circRNA‐007371 was upregulated in fibrotic livers and was resistant to RNase R.

Unlike linear RNA, circRNA can form a covalently closed continuous loop by back‐splicing.^19^ To verify the loop formation of circRNA‐007371, divergent primers, which span the circRNA junction, and convergent primers were applied to amplify murine hepatic cDNA and genomic DNA (gDNA), followed by agarose gel electrophoresis to identify the PCR products. The convergent primers, not the divergent primers, amplified the linear RNA *Gapdh* in both cDNA and gDNA (Figure 4H). However, both divergent and convergent primers of circRNA‐007371 could amplify from cDNA (Figure 4H). Only convergent primers, not divergent primers of circRNA‐007371, could amplify from gDNA (Figure 4H). Sanger sequencing was then applied in amplified products obtained with divergent primers, and a back‐spliced junction of circRNA‐007371 was validated (Figure 4I). In summary, circRNA‐007371 is a circRNA.

### 3.5 Angiogenic role of circRNA‐007371 in EOMA endothelial cells

**FIGURE 5:**
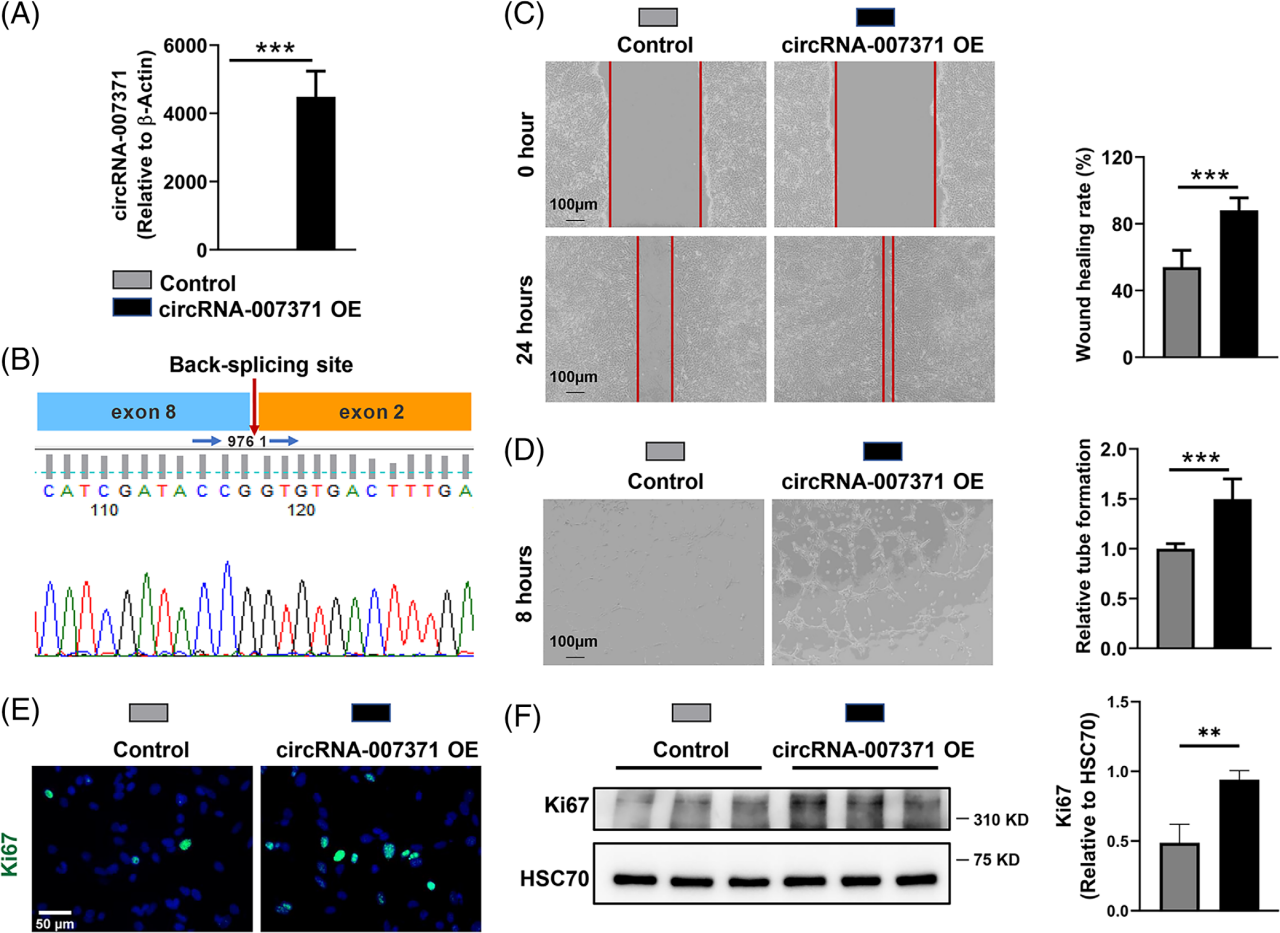
Angiogenic role of circRNA‐007371 in EOMA endothelial cells. EOMA murine endothelial cells were transfected with empty plasmid or circRNA‐007371 overexpressing plasmid. (A) The overexpression of circRNA‐007371 in EOMA cells was determined by qPCR. (B) The back‐spliced junction of circRNA‐007371 was analysed by Sanger sequencing for PCR products obtained with divergent primers in circRNA‐007371 overexpressing EOMA cells. (C) The wound healing assay was used to assess cell migration in EOMA cells. (D) The tube formation assay was used to assess the ability of EOMA cells to form new vessels. (E, F) The cell proliferation of EOMA cells was analysed for Ki67 by immunofluorescence (E) and Western blot (F). *n* = 3/group. **p < 0.01; ***p < 0.001.

circRNAs play crucial roles in the progression of liver diseases^19^ and angiogenesis in HCC.^27^, ^28^ We have verified that increased angiogenesis is accomplished with liver fibrosis. However, whether circRNA‐007371 can regulate angiogenesis remains elusive. Endothelial cell migration, tube formation, and cell proliferation are essential to angiogenesis.^37^ Thus, in vitro, EOMA endothelial cells were transfected with empty plasmid or circRNA‐007371 overexpressing plasmid, then cell migration, tube formation, and cell proliferation were determined. The upregulation of circRNA‐007371 after circRNA‐007371 plasmid transfection was validated by qPCR (Figure 5A). After circRNA‐007371 plasmid transfection, Sanger sequencing with a divergent primer was first applied, and the same back‐spliced junction of circRNA‐007371 was validated (Figure 5B). Compared to the control cells, circRNA‐007371 overexpression promoted cell migration as determined by wound healing assay (Figure 5C). Increased new blood vessel formation and capillary length induced by circRNA‐007371 overexpression in EOMA endothelial cells were also observed (Figure 5D). Furthermore, compared to control cells, increased cell proliferation in circRNA‐007371 overexpressing EOMA cells was determined by IF and WB of Ki67 (Figure 5E,F). In summary, circRNA‐007371 overexpression promotes angiogenesis.

### 3.6 The underlying mechanism of circRNA‐007331 as a miRNA sponge

**FIGURE 6:**
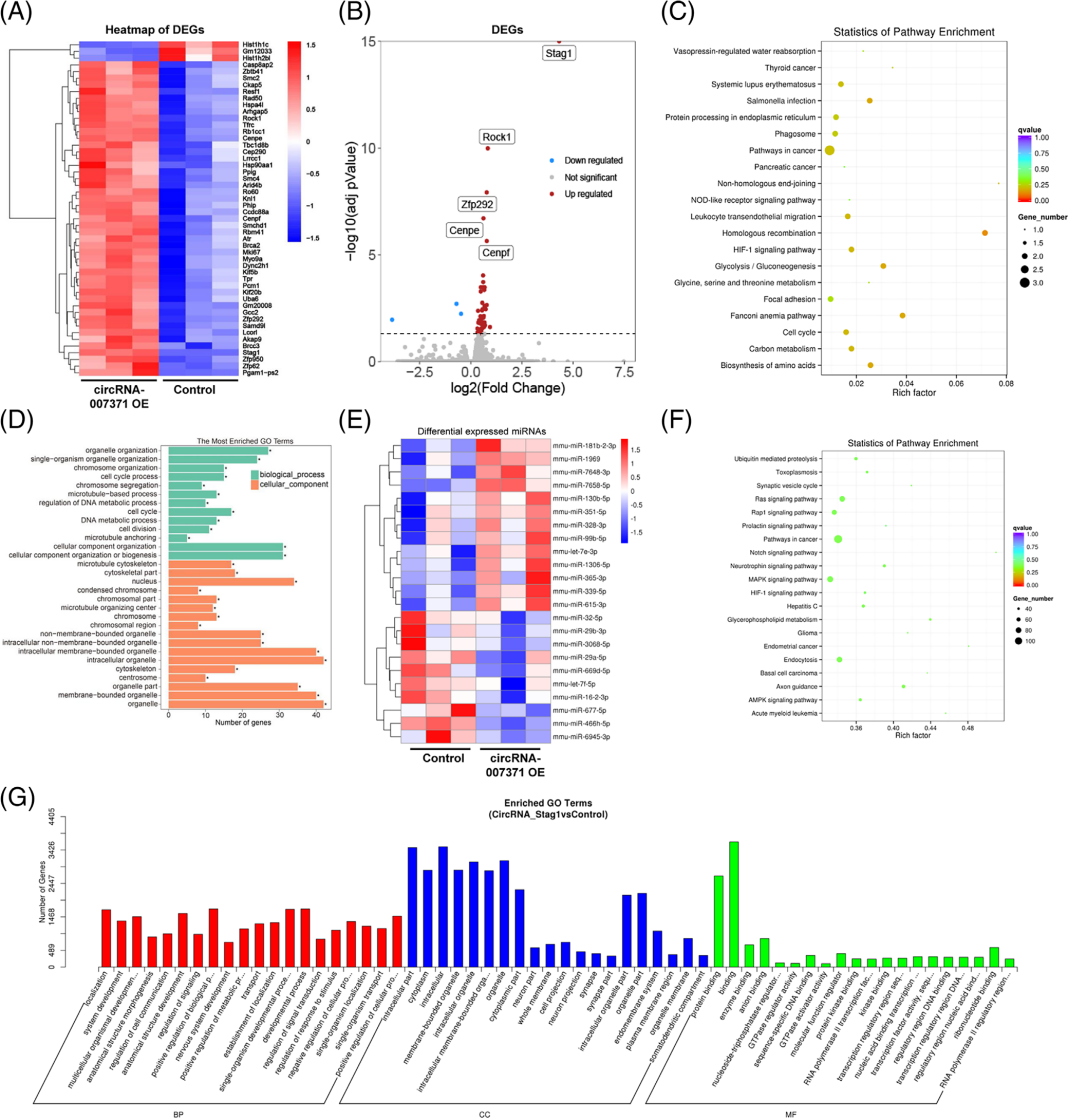
The underlying mechanism of circRNA‐007331 as a microRNA (miRNA) sponge. (A–D) EOMA endothelial cells transfected with the empty plasmid or circRNA‐007371 overexpressing plasmid were analysed by RNA sequencing (*n* = 3/group). The heatmap (A) and volcano plots (B) showed the differentially expressed genes (DEGs). The Kyoto Encyclopedia of Genes and Genomes (KEGG) pathway (C) and Gene Ontology (GO) analysis (D) of DEGs were displayed. (E–G) EOMA endothelial cells transfected with the empty plasmid or circRNA‐007371 overexpressing plasmid were analysed by miRNA sequencing (*n* = 3/group). The differentially expressed miRNAs (DEmiRNAs) were shown in the heatmap (E). The target genes of DEmiRNAs were assigned to the KEGG pathway (F) and GO analysis (G).

As miRNA sponges or ceRNAs, circRNAs can abrogate the target gene inhibition effects of miRNAs.^19^, ^21^, ^22^ Given that upregulated circRNA‐007371 has multiple miRNA binding sites, we examined whether upregulated circRNA‐007371 regulates angiogenesis by sponging miRNA. RNA sequencing was utilized for EOMA endothelial cells transfected with empty plasmid or circRNA‐007371 overexpressing plasmid in vitro. Compared with control cells, 47 upregulated differentially expressed genes (DEGs) and 3 downregulated DEGs were found after circRNA‐007371 overexpression (Figure 6A,B). Of note, *Stag1*, the parent gene of circRNA‐007371, was the most upregulated DEG (Figure 6A,B). In addition, the KEGG pathway and GO analysis of DEGs are displayed (Figure 6C,D). In the KEGG pathway analysis, circRNA‐007371 promoted the activation of angiogenesis‐related signalling pathways, including the phagosome, HIF1, glycolysis, focal adhesion, and cell cycle signalling pathways (Figure 6C). In addition, GO analysis indicated that circRNA‐007371 enhanced the organization of organelles and chromosomes, the cell cycle, and cell division (Figure 6D).

To explore the miRNA sponge or ceRNA mechanism of circRNA‐007371, miRNA sequencing was applied to EOMA endothelial cells transfected with empty plasmid or circRNA‐007371 overexpressing plasmid in vitro. Compared to the control cells, 13 upregulated and 10 downregulated DEmiRNAs were revealed after circRNA‐007371 overexpression, including the well‐defined miR‐29b, miR‐29a, miR‐16, miR‐32, miR‐99b, and miR‐130b (Figure 6E). The target genes of these 23 DEmiRNAs were then assigned to KEGG and GO pathway analyses (Figure 6F,G). In the KEGG pathway analysis, overexpression of circRNA‐007371 participated in the regulation of angiogenesis‐related signalling pathways, including the Ras, Rap1, MAPK, HIF, AMPK, and endocytosis signalling pathways (Figure 6F). In addition, GO analysis indicated that circRNA‐007371 was involved in cell communication, GTPase regulation, and transcription factor activity (Figure 6G). In summary, the angiogenic role of circRNA‐007371 might promote liver fibrosis via miRNA sponges or ceRNA mechanisms.

### 3.7 Involvement of the STAG1–HIF1α signalling pathway in the angiogenic role of circRNA‐007371

**FIGURE 7:**
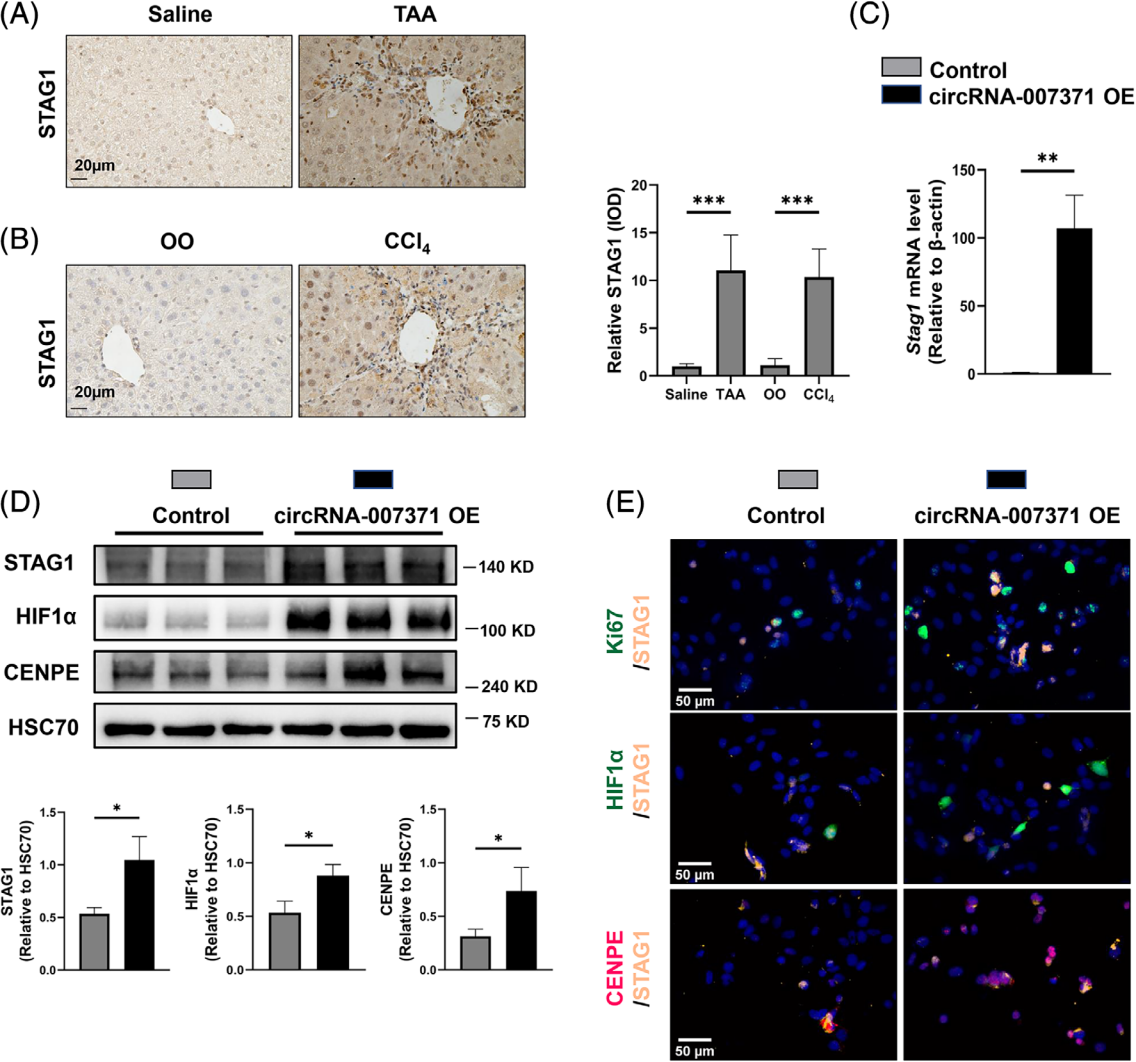
Involvement of the STAG1–HIF1α signalling pathway in the angiogenic role of circRNA‐007371. (A, B) The protein level of STAG1 in murine livers was analysed by immunohistochemical staining in the thioacetamide (TAA)‐ (A) or carbon tetrachloride (CCl_4_)‐induced murine fibrotic models (B). (C–E) EOMA endothelial cells transfected with the empty plasmid or circRNA‐007371 overexpressing plasmid. The mRNA level of *Stag1* in EOMA cells was determined by qPCR (C). The protein levels of STAG1, HIF1α, and CENPE were determined by Western blot (D). Colocalization of Ki67/STAG1, HIF1α/STAG1, and CENPE/STAG1 in EOMA cells was determined by immunofluorescence (E), *n* = 3/group. *p < 0.05; **p < 0.01; ***p < 0.001.

STAG1 is a subunit of the cohesin complex that affects genome organization, sister chromatid cohesion, and gene expression,^38^ and *Stag1* is the parent gene of circRNA‐007371 (Figure 4A). Consistent with the increased circRNA‐007371 in murine fibrotic livers (Figure 4G), the protein level of STAG1 was raised in TAA‐ and CCl_4_‐induced murine fibrotic livers compared to control livers (Figure 7A,B). Meanwhile, the mRNA and protein levels of STAG1 in circRNA‐007371 overexpressing EOMA cells were also significantly increased (Figure 7C–E).

Subsequently, the molecules and signalling pathways enriched in circRNA‐007371 overexpressing EOMA endothelial cells were validated by IF and WB. HIF1α is the crucial regulator for angiogenesis, and CENPE plays a key role in cell proliferation.^39^ Compared to control cells, increased protein levels of HIF1α and CENPE determined by WB were observed in circRNA‐007371 overexpressing EOMA cells (Figure 7D). Cell proliferation is an essential mechanism of angiogenesis. Whether STAG1, HIF1α, and CENPE involve angiogenesis via regulating cell proliferation remains unclear. Increased colocalization of Ki67/STAG1, HIF1α/STAG1, and CENPE/STAG1 was observed in circRNA‐007371 overexpressing EOMA cells compared to control cells (Figure 7E). In summary, the STAG1–HIF1α signalling pathway may contribute to the angiogenic role of circRNA‐007371 in liver fibrosis.

## 4 DISCUSSION

**FIGURE 8:**
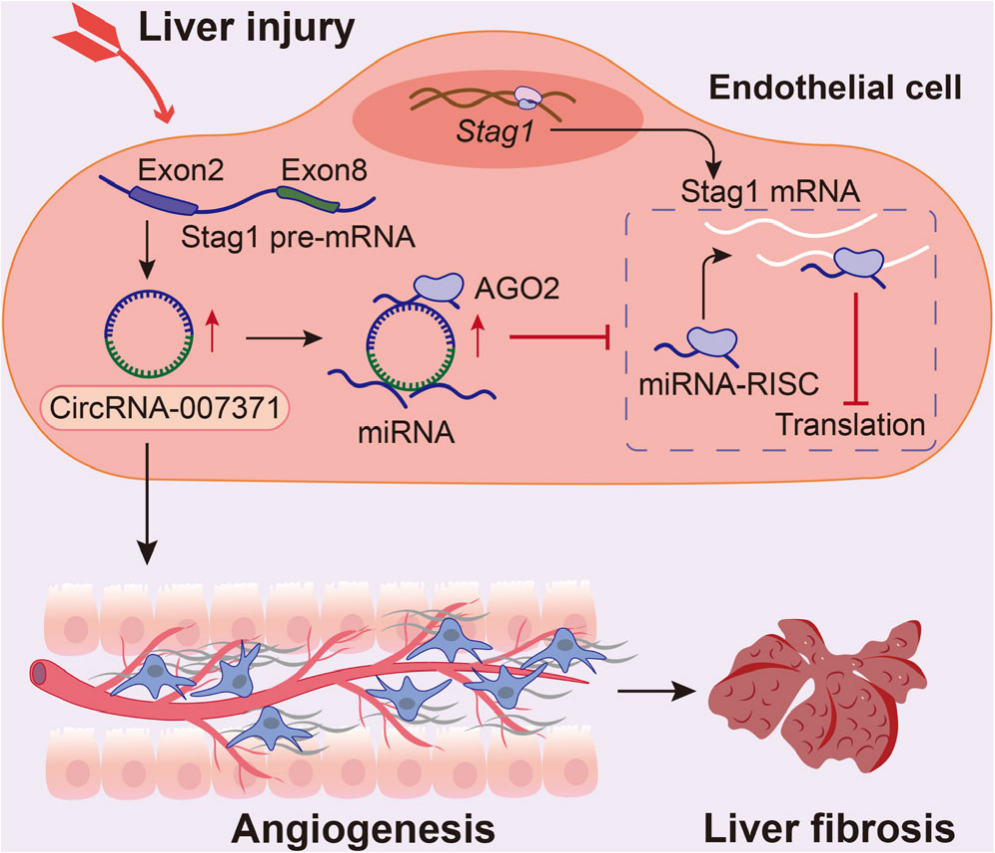
Proposed mechanism. Increased angiogenesis is accomplished with liver fibrosis. circRNA‐007371 enhances angiogenesis in liver fibrosis via increasing STAG1 in a miRNA sponge mechanism.

Liver cirrhosis and CLD lead to a high disease burden, and in‐depth mechanisms are urgently needed to explore effective therapeutic targets.^4^ circRNAs are pivotal regulators of gene expression during the pathogenesis of HCC, whereas the role and mechanism of circRNAs in liver cirrhosis remain elusive. We explored and verified circRNA‐007371 as a new circRNA involved in the pathogenesis of liver fibrosis. We demonstrated a new mechanism by which circRNA‐007371 promoted angiogenesis via the STAG1–HIF1α signalling pathway as a miRNA sponge (Figure 8).

In liver fibrosis, most research on angiogenesis focuses on the VEGF–HIF signalling pathway in LSECs or the crosstalk of LSECs with HSCs.^13^, ^15^, ^40^ When hypoxia occurs in a tissue, HIF1α is upregulated,^41^ which induces the increased expression of VEGFA and angiopoietin 2 to promote angiogenesis.^37^ Consistently, increased angiogenesis and VEGFA/VEGFR2 axis were confirmed in TAA‐ and CCl_4_‐induced murine fibrotic livers. Recently, DNA nanoparticles have been used to regulate angiogenesis in some diseases.^42^, ^43^ However, whether other regulators can promote angiogenesis in the context of liver fibrosis remains unclear. circRNAs are a subtype of noncoding RNA with a covalently closed continuous loop.^19^ It has been demonstrated that circRNAs can induce angiogenesis in HCC.^27^, ^28^ However, the function and mechanism of circRNAs in angiogenesis in liver fibrosis have not been well characterized. By screening with circRNA microarray and AGO2‐RIP sequencing, circRNA‐007371 was selected as the target circRNA in this study. circRNA‐007371 overexpression promotes angiogenesis in EOMA cells. It seems that circRNA‐007371 might involve in the pathogenesis of liver cirrhosis by enhancing angiogenesis.

The covalent loop of circRNA is formed by back‐splicing from parent linear pre‐mRNA.^19^ circRNA‐007371 is a 976 bp novel circRNA by back‐splicing from exon 2 and exon 8 of *Stag1*. Similar to other well‐studied circRNAs with the advantages of stability, circRNA‐007371 is resistant to RNase R. However, as a novel circRNA, it is a great challenge to explore the functions of circRNA‐007371 in liver cirrhosis. circRNAs, as miRNA sponges or ceRNAs, can compete with the target sites of miRNA–RISC, further relieving the parent gene suppression of miRNAs to regulate the gene expression process.^21^, ^22^ The miRNA sponge role of circRNAs has been illustrated in liver fibrosis by controlling HSC activation^24^ and quiescence.^25^, ^26^ Whether circRNA‐007371 also promotes angiogenesis as a miRNA sponge needs to be determined. In this study, circRNA‐007371 was enriched in AGO2‐RIP sequencing of the murine fibrotic livers, which means that circRNA‐007371 combined with miRNA–RISC to serve as a miRNA sponge. Consistently, circRNA‐007371 overexpression induced the upregulation of *Stag1* and the disturbance of several miRNAs in EOMA endothelial cells. STAG1 contributes to genome organization, sister chromatid cohesion, and gene expression.^38^ In this study, STAG1 was accomplished with circRNA‐007371 overexpression, angiogenesis, and cell proliferation. Angiogenesis is regulated by several signalling pathways, including the MAPK, Notch, and HIF1α signalling pathways.^13^, ^15^ The HIF1α signalling pathway was significantly enriched in the KEGG analysis of mRNA sequencing and miRNA sequencing by circRNA‐007371 overexpression in EOMA endothelial cells. The protein level of HIF1α and colocalization of STAG1/HIF1α were validated in circRNA‐007371 overexpressing EOMA cells. These results confirmed that circRNA‐007371 could regulate angiogenesis via the STAG1–HIF1α signalling pathway. Collectively, the current study displayed that the circRNA‐007371–STAG1–HIF1α axis may regulate angiogenesis during liver fibrosis.

In conclusion, circRNA‐007371 enhances angiogenesis via a miRNA sponge mechanism in liver fibrosis. The STAG1–HIF1α signalling pathway might contribute to the proangiogenic effect of circRNA‐007371. The strategy targeting the inhibition of circRNA‐007371 might provide a novel therapeutic approach for treating liver cirrhosis.

## AUTHOR CONTRIBUTIONS

Jinhang Gao, Zhiyin Huang, and Chengwei Tang conceived and supervised the study. Chong Zhao, Shuaijie Qian, Yang Tai, and Yangkun Guo performed experiments and analysed the data. Chong Zhao, Shuaijie Qian, Zhiyin Huang, and Jinhang Gao wrote the manuscript with input from all the authors.

## CONFLICT OF INTEREST STATEMENT

The authors declare no conflict of interest.

## Supporting information


**DATA S1.** Supporting Inforamtion

## Data Availability

The high‐throughput data are deposited in GSE218581. All the data are available from the corresponding author Jinhang Gao (gao.jinhang@scu.edu.cn) under reasonable request.
