# Supplementary material for: Proangiogenic role of circRNA‐007371 in liver fibrosis
Source: Cell Prolif. 2023 Feb 28;56(6):e13432. doi: 10.1111/cpr.13432 (PMC10280135; doi:10.1111/cpr.13432)
Supplement: Supplementary file 1 — DATA S1. Supporting Inforamtion [file CPR-56-e13432-s001.docx]

**Supporting Information**

**Proangiogenic role of circRNA-007371 in liver fibrosis**

Chong Zhao^1,2#^, Shuaijie Qian^1,2#^, Yang Tai^1,2^, Yangkun Guo^1,2^, Chengwei Tang^1,2^, Zhiyin Huang^2^*, Jinhang Gao^1,2^*

*1 Lab of Gastroenterology and Hepatology, West China Hospital, Sichuan University, Chengdu, China.*

*2 Department of Gastroenterology, West China Hospital, Sichuan University, Chengdu, China.*

**Supporting Methods**

**Histological study**

4% paraformaldehyde (PFA)-fixed paraffin liver sections of 5 μm thickness were stained with hematoxylin and eosin (H&E) and Sirius red according to the standard protocol. Five images per section were randomly captured using the CX41 histology microscope (Olympus Corporation, Tokyo, Japan) with the DP72 digital camera (Olympus Corporation). Percentages of fibrotic and vascular areas were analyzed using ImageJ software (National Institutes of Health, Bethesda, MD, USA).

**Immunohistochemical staining (IHC)**

The xylene and graded ethanol dilutions were used to deparaffinized and rehydrate for PFA-fixed paraffin liver sections of 5 μm thickness. After heat-induced antigen retrieval in sodium citrate buffer (10 mM, pH=6.0), liver sections were incubated with 3% H_2_O_2_ for 15 minutes, blocked in 10% goat serum for 30 minutes, then incubated with primary antibody at 4°C overnight, followed by detection with 3,3’-diaminobenzidine (DAB, ZSGB-BIO). Primary antibodies used include: VEGFA (1:100, Abcam #ab1316), VEGFR2 (1:1000, Abcam #ab233693), CD31 (1:200, Abcam #ab222783), vWF (1:500, ABclonal #A21054), QKI (1:500, Abcam #ab126742), and STAG1 (1:500, Abcam #ab4457). A two-step biotin-streptavidin horseradish peroxidase detection kit (ZSGB-BIO, Beijing, China) was then applied. After being counterstained with hematoxylin, the slides were detected under a histology microscope (CX41) with a digital camera (DP72). The positive areas of the murine liver were analyzed using ImageJ software.

**Immunofluorescence staining (IF)**

The xylene and graded ethanol dilutions were used to deparaffinized and rehydrate for PFA-fixed paraffin liver sections of 5 μm thickness, followed by heat-induced antigen retrieval in sodium citrate buffer (10 mM, pH=6.0). The EOMA cells were fixed by 4% PFA. Liver sections or EOMA cells were blocked in 10% goat serum for 30 minutes, then incubated with collagen I (1:200, SouthernBiotech #1310-01), αSMA (1:200, Abcam #ab124964), LYVE-1 (1:400, R&D Systems #AF2125), Ki67 (1:200, Abcam #ab15580), STAG1 (1:500, Abcam #ab4457), HIF1α (1:200, CST #D1S7W), CENPE (1:200, Santa Cruz Biotechnology #sc376685) at 4°C overnight, followed being incubated with the Alexa Fluor secondary antibodies (1:100, Abcam #ab150062, #150130, #150105, #150110) for 45 minutes. The slides were detected under a histology microscope (CX41) with a digital camera (DP72). The positive areas of the murine liver were analyzed using ImageJ software.

**Western blot**

The protein extraction kit (Keygen Biotech, Nanjing, China) was utilized to extract the proteins from liver tissues or cells. Fifty μg of protein was loaded onto SDS‒PAGE (Beyotime, Jiangsu, China) and transferred to PVDF membranes (Millipore, Billerica, MA, USA). 5% nonfat milk was utilized to seal membranes, followed by a primary antibody was used to incubate overnight at 4℃. Primary antibodies used include: collagen I (1:1000, Abcam# ab260043); αSMA (1:2000, Abcam #ab124964), Ki67 (1:200, Abcam #ab15580), STAG1 (1:500, Abcam #ab4457), HIF1α (1:200, CST #D1S7W), CENPE (1:200, Santa Cruz Biotechnology #sc376685), HSC70 (1:1000, Santa Cruz Biotechnology #SC-7298). Subsequently, the blots were hatched with secondary antibodies (1:10000, ZSGB-BIO). The blots were visualized using an enhanced chemiluminescence detection kit (Beyotime #P0018AM). Protein levels were normalized against HSC70. The result analysis was performed using ImageJ software.

**CircRNA microarray and data analysis**

Five liver tissues from the TAA group and 5 liver tissues from the saline group were sent to circRNA microarray. The circRNA microarray procedure was accomplished by Shanghai KangCheng Biotech (Shanghai, China). Based on the manufacturer’s instructions of Arraystar, total RNAs from liver tissues were isolated using TRIzol reagent. After fluorescent cRNAs transcribing from total RNAs, the labeled cRNAs were hybridized into an Arraystar Mouse circRNA Array (6x7K, Arraystar). The circRNA Array was detected by Axon GenePix 4000B microarray scanner. The R package was used to analyze the results. Differentially expressed (DE) circRNAs between 2 groups were identified by fold-changes >2 and *p*-value <0.05. Heatmaps were generated to visualize gene expression patterns. Kyoto Encyclopedia of Genes and Genomes (KEGG) analysis and Gene Ontology (GO) analysis of the DEcircRNAs were utilized. The raw data of the circRNA microarray could be found in the NCBI GEO (GSE218574).

**Anti-AGO2 [RNA immunoprecipitation (RIP) sequencing and data analysis](https://www.abcam.com/epigenetics/rna-immunoprecipitation-rip-protocol)**

Anti-AGO2-RIP followed by RNA sequencing (AGO2-RIP sequencing) was used to explore the expression of RNAs that bind to AGO2. Liver tissues from the TAA group were utilized for AGO2-RIP sequencing. AGO2-RIP sequencing was accomplished by Shanghai Cloud-Seq Biotech (Shanghai, China) according to the RNA immunoprecipitation (RIP) protocol recommended by Millipore. In brief, the lysed liver tissues were incubated with magnetic beads coupled with anti-immunoglobulin G (IgG) antibodies and anti-AGO2 antibodies (Abcam #Ab156870) overnight at 4°C.

TRIzol reagent was utilized to extract RNA from the AGO2-RNA complex. The RNA libraries were established, and RNA sequencing was then implemented with HiSeq 4000 Sequencer (Illumina). STAR software (v2.5.1b) was used to map quality-filtered reads to the genome/transcriptome after removing low-quality reads. After detecting and identifying circRNAs with DCC software (v0.4.4), the R package was utilized to analyze the results. DEcircRNAs were identified by *p*-value <0.05. The raw data of AGO2-RIP sequencing could be found in the NCBI GEO (GSE218577).

**mRNA sequencing and data analysis**

The control and circRNA-007371 overexpressing EOMA cells were subjected to RNA sequencing performed by Novogene (Beijing, China). Briefly, EOMA cells were lysed by TRIzol reagent to obtain RNA. After mRNA libraries were established, NovaSeq 6000 platform (Illumina) was applied to perform RNA sequencing. HISAT2 (v2.0.4) was utilized to map quality-filtered reads to the reference genome. HTSeq (v0.6.1) was utilized to get read counts for each gene. Differentially expressed genes (DEGs) were identified by *p*-value <0.05. KEGG and GO analysis of the DEGs were implemented. The raw data of RNA sequencing were deposited in the NCBI GEO (GSE218578).

**MicroRNA sequencing (miRNA sequencing) and data analysis**

Control and circRNA-007371 overexpressing EOMA cells were utilized for miRNA sequencing performed by Novogene (Beijing, China). EOMA cells were lysed by TRIzol reagent. The miRNA libraries were prepared, and miRNA sequencing was then performed on HiSeq 2500/2000 platform (Illumina). HISAT2 (v2.0.4) was utilized to map quality-filtered reads to reference the genome. Bowtie was used to obtain read counts for each gene. Differentially expressed (DE) miRNAs were identified by *p*-value <0.05. KEGG and GO analysis of the DEmiRNA target genes were implemented with *p*-value <0.05. The raw data were deposited in the NCBI GEO (GSE218579).

**Quantitative RT‒PCR (qPCR) and agarose gel electrophoresis**

Liver tissue or EOMA cells were lysed by RNA Isolation Kit (Foregene #RE-03014, Chengdu, China) to get total RNA. After reverse transcription by a reverse first-strand cDNA synthesis kit (Thermo Scientific #K1622) with 1 μg RNA, qPCR was performed using SYBR Green qPCR Master Mix (Bimake #B21202). The expression of circRNA or mRNA was normalized to *β-actin*.

After the PCR process, agarose gel electrophoresis was used to detect the products. Briefly, PCR products were resolved by an agarose gel (Invitrogen, #75510019) under 100 V, followed by visualization with the UV mode of Chemiscope 6100. The primers are listed in Supporting Table S1.

**RNase R treatment assay**

RNA from TAA-induced fibrotic liver tissues was treated with RNase R (Abcam #ab286929) or vehicle for 30 minutes and then analyzed by qPCR or agarose gel electrophoresis.

**Cell culture and circRNA-007371 overexpression**

EOMA cells were got from the American Type Culture Collection. EOMA cells were cultured in Dulbecco's modified Eagle's medium (DMEM) at 5% CO_2_ and 37°C. EOMA cells were transfected with circRNA-007371 overexpressing plasmid (circRNA-007371-pLC5-ciR, Geneseed Biotech, Guangzhou, China) and its control plasmid (Empty- pLC5-ciR, Geneseed Biotech) by lipofectamine 2000 (Invitrogen #11668500). Eventually, the cells were assigned for the corresponding tests. The overexpression of circRNA-007371 was confirmed by qRT‒PCR and Sanger DNA sequencing.

**Wound healing assay**

EOMA were sowed into 6 well plates with 5×10^4^ cells/well. The 200 μL micropipette tip was used to scrap a confluent cell monolayer and form a wound in 2 mm width. The culture media was changed, and cells were incubated to allow for healing. At 0 hours and 24 hours after wound formation, images were scanned using an inverted microscope, and the scratch-wound area was quantified by ImageJ. The wound-healing rate was estimated following the formula: (width 0 hour-width 24 hours)/width 0 hour×100%.

**Tube formation assay**

100 μL of Matrigel per well, which melted at 4°C overnight, was vertically plated into 24-well plates at 37°C for 30 minutes to solidify the Matrigel. EOMA were then seeded with 5×10^3^ cells/well. After culturing at 37°C for 8 hours, images were obtained under a phase contrast microscope. The capillary length was analyzed by ImageJ.

**Supporting Table and Figure**

**Supporting Table S1. The primer sequences**

| **Gene** | **Sequence-forward** | **Sequence-reverse** | **Product (bp)** |
| --- | --- | --- | --- |
| CircRNA-007371-divergent | GACTGGAGTTACTACTTCAGA | AGTAATCATTGCTGGAGAA | 188 |
| CircRNA-007371-convergent | AATGAAACTACTGCCCACT | CTCTTCCACGACCTGCT | 175 |
| CircRNA-007371-qPCR | tgttcatcgataccggtgtg | CAGAGTGGGCAGTAGTTTCA | 149 |
| *Gapdh-*divergent | GTCGTGGAGTCTACTGGTGTC | ATTTGCCGTGAGTGGAGTC | 190 |
| *Gapdh-*convergent | GACTCCACTCACGGCAAAT | GACACCAGTAGACTCCACGAC | 190 |
| *Stag1-qPCR* | CTGCCCACTCTGATGCTG | GCTCTTCCACGACCTGCT | 166 |
| *β-actin-qPCR* | TGACGTTGACATCCGTAAAG | GAGGAGCAATGATCTTGATCT | 143 |

**Supporting Figure**


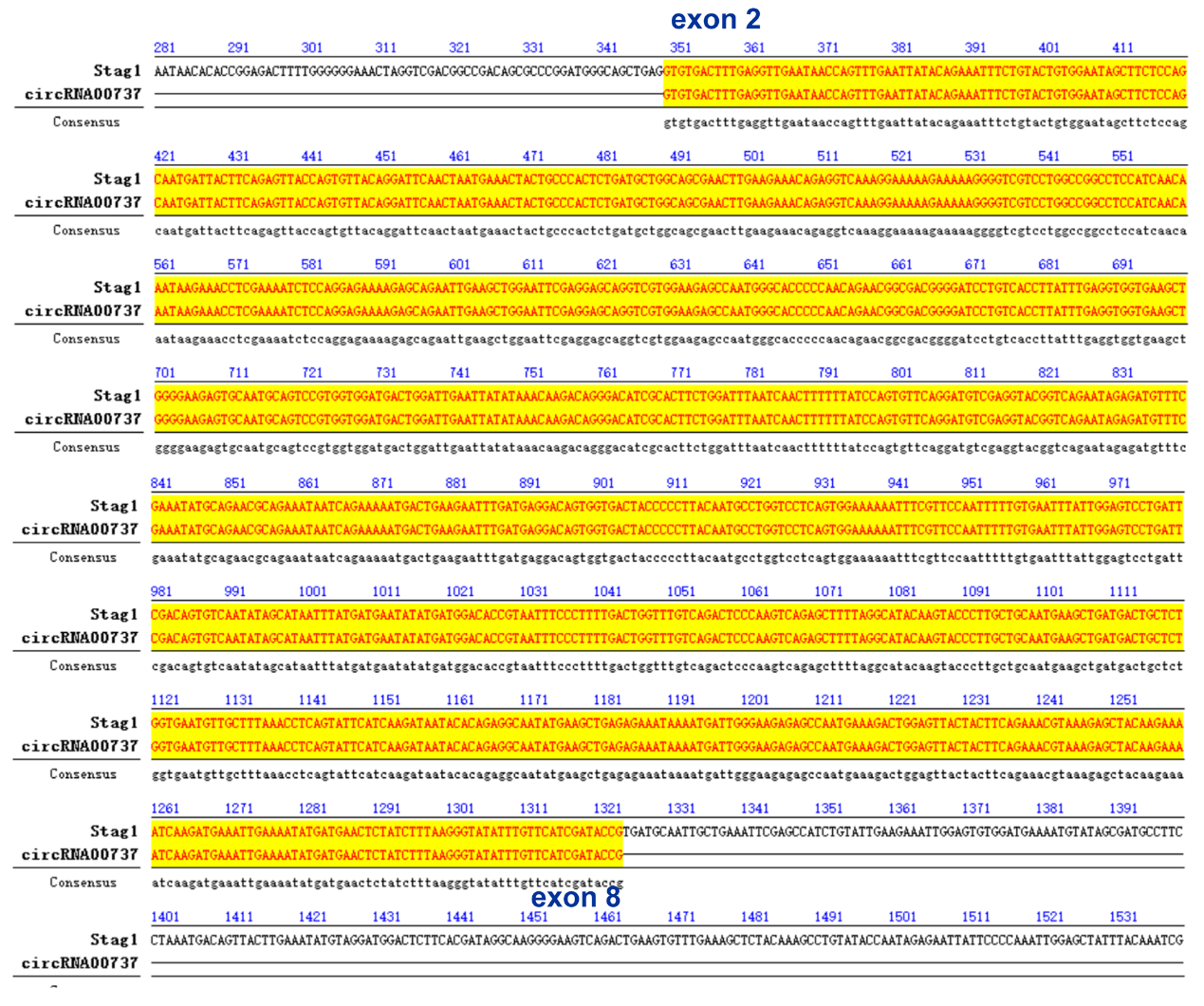


**Supporting Figure S1. Sequence comparison of circRNA-007371 and Stag1 using Jellyfish software**
